# Oral Zinc Supplementation Reduces the Erythropoietin Responsiveness Index in Patients on Hemodialysis

**DOI:** 10.3390/nu7053783

**Published:** 2015-05-15

**Authors:** Hiroki Kobayashi, Masanori Abe, Kazuyoshi Okada, Ritsukou Tei, Noriaki Maruyama, Fumito Kikuchi, Terumi Higuchi, Masayoshi Soma

**Affiliations:** 1 Division of Nephrology, Hypertension and Endocrinology, Department of Internal Medicine, Nihon University School of Medicine, 30-1 Oyaguchi Kami-chou, Itabashi-ku, Tokyo 173-8610, Japan; E-Mails: kobayashihiroki2@gmail.com (H.K.); kokada@med.nihon-u.ac.jp (K.O.); haru_li_huang@yahoo.co.jp (R.T.); maruyama.noriaki@nihon-u.ac.jp (N.M.); souma.masayoshi@nihon-u.ac.jp (M.S.); 2 Department of Nephrology, Meirikai Chuo General Hospital, 3-2-11, Higashijujou, Kita-ku, 114-0001 Tokyo, Japan; E-Mail: kikuchi5308@air.ocn.ne.jp; 3 Department of Nephrology, Keiai Hospital, 3-10-6, Mukaihara, Itabashi-ku, 173-0036 Tokyo, Japan; E-Mail: thiguchi@keiai-hospital.jp

**Keywords:** erythropoietin responsiveness index, erythropoiesis stimulating agent, hemodialysis, renal anemia, zinc deficiency

## Abstract

Background: In hemodialysis (HD) patients, zinc depletion caused by inadequate intake, malabsorption, and removal by HD treatment leads to erythropoiesis-stimulating agent (ESA) hyporesponsiveness. This study investigated the effects of zinc supplementation in HD patients with zinc deficiency on changes in the erythropoietin responsiveness index (ERI). Methods: Patients on HD with low serum zinc levels (<65 μg/dL) were randomly assigned to two groups: The polaprezinc group (who received daily polaprezinc, containing 34 mg/day of zinc) (*n* = 35) and the control group (no supplementation) (*n* = 35) for 12 months. All the 70 patients had been taking epoetin alpha as treatment for renal anemia. ERI was measured with the following equation: Weekly ESA dose (units)/dry weight (kg)/hemoglobin (g/dL). Results: There were no significant changes in hemoglobin levels within groups or between the control and polaprezinc groups during the study period. Although reticulocyte counts were increased immediately after zinc supplementation, this change was transient. Serum zinc levels were significantly increased and serum copper levels were significantly decreased in the polaprezinc group after three months; this persisted throughout the study period. Although there was no significant change in the serum iron or transferrin saturation levels in the polaprezinc group during the study period, serum ferritin levels significantly decreased following polaprezinc treatment. Further, in the polaprezinc group, ESA dosage and ERI were significantly decreased at 10 months and nine months, respectively, as compared with the baseline value. Multiple stepwise regression analysis revealed that the change in the serum zinc level was an independent predictor of lowered ERI. Conclusions: Zinc supplementation reduces ERI in patients undergoing HD and may be a novel therapeutic strategy for patients with renal anemia and low serum zinc levels.

## 1. Introduction

The main therapeutic options for anemia of end-stage renal disease (ESRD) include treatment with an erythropoiesis-stimulating agent (ESA) and iron supplementation. An important issue in treating this type of anemia is that the ESA dosage required to achieve target hemoglobin concentration varies widely among ESRD patients. Although some factors have been reported to contribute to ESA hyporesponsiveness, including iron deficiency, chronic blood loss, inflammation, vitamin deficiencies, and hyperparathyroidism [1,2,3,4], few studies have focused on other factors affecting hyporesponsiveness.

Patients undergoing hemodialysis (HD) have been found to have low serum concentrations of zinc due to zinc removal during HD and decreased serum albumin levels as well as inadequate dietary intake and reduced gastrointestinal absorption of zinc [5,6]. Serum zinc levels can also be reduced by increased expression of intracellular metallothioneins following oxidative stress or up-regulation of zinc-importing proteins by pro-inflammatory cytokines [7]. The relationship between zinc deficiency and renal anemia in dialysis patients receiving ESA therapy has not been well studied. In this study, we investigated the effects of zinc supplementation in HD patients with zinc deficiency on changes in the erythropoietin responsiveness index (ERI) by evaluating changes in the levels of hemoglobin, zinc, iron, ferritin, and ESA dosage.

## 2. Subjects and Methods

### 2.1. Study Participants

This prospective, open-label, randomized, parallel-group, multi-center trial examined 70 HD patients who were randomly assigned to the oral polaprezinc therapy group (*n* = 35) or control group (*n* = 35, no supplementation). Patients on polaprezinc treatment (administered at 150 mg, containing zinc at 34 mg/day) and controls were monitored for 12 months. Subjects were randomly assigned to the two groups prior to the start of the study. An independent investigator with no previous knowledge of the subjects before commencement of the trial monitored randomization of subject entry order. Dynamic balancing randomization was carried out based on age, sex, HD duration, hemoglobin level, ESA dosage, and presence or absence of diabetes mellitus. Thus, we ensured that there were no significant differences in baseline characteristics between the groups. The details of the assignment were then given to three independent investigators. The study protocol was approved by the Ethics Committee of our hospital and all patients gave written informed consent (Clinical Trial Registration number: UMIN000017032). The study protocol was designed in accordance with the Declaration of Helsinki. All patients were treated with HD therapy three times weekly in 4-h sessions at the blood purification unit of our hospital; patients treated between January 2013 and December 2014 were considered for this study.

Enrollment criteria were as follows: (1) age ≥20 years or ≤85 years, (2) on HD for at least six months, and (3) zinc deficiency defined as a serum zinc level <65 μg/dL. Exclusion criteria were as follows: (1) age of <20 years or >85 years; (2) recent infection, cancer, or drug/alcohol abuse; (3) treatment with steroids or immunosuppressants; (4) currently hospitalized; (5) previous history of blood transfusion within at least six months before the study; or (6) recent hospitalization or unwillingness to participate in the study. Patients continued their regular medications such as anti-hypertensive agents, phosphate binders, and lipid-lowering agents during the study period. Patients were regularly given dietary guidance, especially those under dietary restrictions (such as for salt and protein intake).

Cardiovascular disease comorbidities were defined as previous ischemic heart disease or aortic disease, conditions requiring percutaneous coronary intervention or surgical revascularization, previous cerebrovascular disease, or peripheral artery disease treated previously or currently.

### 2.2. Study Evaluations

Blood cell counts, serum creatinine, blood urea nitrogen, electrolytes, uric acid, serum iron, total iron binding capacity, serum ferritin, total cholesterol, high-density lipoprotein cholesterol, triglyceride, total protein, and albumin levels were measured by routine clinical chemistry procedures using commercial kits. High-sensitivity C-reactive protein (hs-CRP) was measured by latex agglutination. Intact parathyroid hormone was measured by radioimmunoassay. Serum zinc concentrations were measured by atomic absorption spectrometry. Blood samples were obtained before the start of an HD session. Kt/V was measured monthly from pre-dialysis and immediate post-dialysis blood urea nitrogen levels using a formal single-compartment model of HD urea kinetics. Blood counts were measured twice monthly, and serum levels of iron-associated parameters, zinc, and copper were measured once every three months.

### 2.3. Anemia Management Protocol

All patients received the same ESA, namely, recombinant human erythropoietin (epoetin alpha). The required ESA dose was determined by the following protocol. According to the Japanese Society for Dialysis Therapy (JSDT) guidelines for renal anemia [1], the target hemoglobin (Hb) level in blood samples collected in the supine position before HD at the beginning of the week was 10–11 g/dL. We measured Hb levels twice monthly during the 12-month treatment period. Based on these results, the protocol was used for ESA dose adjustments.

When the hemoglobin level exceeded 12.5 g/dL, ESA was discontinued for two weeks. The dose was then reduced by 25%–50% and resumed when the hemoglobin level decreased to <12.0 g/dL. For hemoglobin levels >11.5 g/dL, the ESA dose was reduced by 25%. No change in dose was made for hemoglobin levels in the range of 10.0–11.5 g/dL. For hemoglobin levels <10.0 g/dL, the ESA dose was increased by 25%–50%. For any decrease in hemoglobin of more than 1 g/dL in a two-week period, the individual’s physician was asked to evaluate them for possible bleeding or hemolysis. ESA treatment was administered intravenously at each HD treatment.

According to the JSDT guidelines, iron deficiency is diagnosed when transferrin saturation (TSAT) is ≤20% and/or serum ferritin level is ≤100 ng/mL [1]. In such cases, there are no contraindications for iron preparations, and cideferron (containing 50 mg of iron) solution is slowly administered via the venous side of the dialysis circuit at the end of dialysis for 13 consecutive sessions.

ERI was defined as the average weekly ESA dose divided by clinical dry weight and average blood hemoglobin (weekly ESA dose (units)/dry weight (kg)/hemoglobin (g/dL)), as described previously [8,9], to normalize the amount of required ESA to the severity of anemia.

### 2.4. HD Procedure

In all patients, the HD procedure was performed using dialyzers containing high-flux membranes (such as polysulfone, polyester-polymer alloy, or cellulose triacetate) at a blood flow rate of 200–250 mL/min and a dialysate flow rate of 500 mL/min. The surface area of the dialyzer membrane was selected according to patient body weight. The glucose concentration of the dialysate was 100 mg/dL. Heparin was administered at 2600–5000 U per 4-h HD session for anticoagulation. The volume of ultrafiltration was maintained on the basis of clinical dry weight during each session.

### 2.5. Statistical Analysis

Results are expressed as mean ± SD. We assessed differences between baseline data and each measurement value using the *t*-test for paired data. The unpaired *t*-test was used to compare baseline mean values between treatment groups. Differences in post-treatment mean values were evaluated using the unpaired Student’s *t*-test for parametric data and the Mann-Whitney test for non-parametric data. Categorical data were compared using repeated-measures analysis of variance. The difference in ERI values between the baseline and end of the study was defined as ΔERI. Spearman’s correlation coefficients were computed to evaluate the relationship between ΔERI and other measured parameters. A multiple linear regression model was built with ΔERI as the dependent variable and measured clinical parameters entered as independent variables. JMP ver. 11 (SAS Institute Inc., Cary, NC, USA) was used for all analyses. Statistical significance was established at *p* < 0.05.

## 3. Results

### 3.1. Baseline Demographic and Clinical Data

Seventy HD patients were enrolled in this trial and randomly allocated to the polaprezinc treatment group or control group (35 patients per group). There were no significant differences in the baseline demographic, metabolic, hemodynamic, anthropometric, or inflammatory variables between the groups (Table 1). Four patients did not complete the assessment or treatment: Two in the polaprezinc group and two in the control group. The other 33 patients in the polaprezinc group and 33 in the control group were included in the final analysis. In the polaprezinc group, subjects were excluded from the final analysis because of transfer to another hospital (*n* = 1) and hospitalization due to heart failure (*n* = 1). In the control group, exclusions were due to hospitalization due to pneumonia (*n* = 1) and hospitalization due to cerebral hemorrhage (*n* = 1). During the study period, angiotensin receptor blocker (ARB) treatment was interrupted in one patient, while the calcium channel blocker (CCB) dose was reduced in two patients and initiated in one patient of the polaprezinc group. In the control group, ARB treatment was initiated in one patient and the CCB dose was increased in two patients. None of the patients required initiation of angiotensin-converting enzyme inhibitor therapy, active vitamin D therapy, proton pump inhibitor therapy, or vitamin C/vitamin E supplementations during the study period.

**Table 1:** Baseline characteristics of the two groups.

Variables	Polaprezinc Group	Control Group	*p* value
*n* (male/female)	35 (21/14)	35 (22/13)	0.811
Age (years)	69 ± 10	69 ± 10	0.797
Hemodialysis duration (mo)	66 ± 45	64 ± 43	0.361
Diabetes mellitus (%)	16 (45.7)	15 (42.8)	0.813
Cardiovascular comorbidity (%)	6 (17.1)	5 (14.2)	0.746
Systolic blood pressure (mmHg)	143 ± 15	144 ± 19	0.210
Diastolic blood pressure (mmHg)	77 ± 14	75 ± 13	0.818
Heart rate (bpm)	74 ± 10	76 ± 10	0.713
Medications (%)			
Active vitamin D therapy	48.5	45.7	0.814
Angiotensin receptor blockers	57.1	60.0	0.811
ACE inhibitors	8.5	5.7	0.648
Calcium channel blockers	51.4	54.2	0.814
Phosphate binders	100	100	1.000
Proton pump inhibitors	85.7	88.5	0.725
Statins	42.8	37.1	0.631
Kt/V	1.33 ± 0.06	1.34 ± 0.06	0.520
Body mass index (kg/m^2^)	21.5 ± 3.5	21.8 ± 3.2	0.715
Hemoglobin (g/dL)	10.8 ± 0.7	11.1 ± 0.8	0.240
Erythropoietin responsiveness index	10.5 ± 5.2	10.2 ± 8.0	0.851
Serum zinc (mg/dL)	53 ± 6	55 ± 5	0.427
Serum creatinine (mg/dL)	8.2 ± 1.3	8.3 ± 1.2	0.831
Serum urea nitrogen (mg/dL)	67 ± 9	78 ± 9	0.376
Serum albumin (g/dL)	3.7 ± 0.3	3.7 ± 0.3	0.623
Total cholesterol (mg/dL)	150 ± 28	151 ± 26	0.492
HDL cholesterol (mg/dL)	49 ± 10	50 ± 11	0.427
Triglycerides (mg/dL)	110 ± 52	112 ± 49	0.422
High sensitivity-CRP (mg/dL)	0.17 ± 0.17	0.18 ± 0.17	0.561
Corrected calcium (mg/dL)	8.7 ± 0.5	8.6 ± 0.4	0.362
Phosphate (mg/dL)	5.7 ± 0.5	5.6 ± 0.5	0.860
Intact PTH (pg/mL)	111 ± 63	92 ± 61	0.104

ACE, angiotensin-converting enzyme; CRP, C-reactive protein; HDL, high-density lipoprotein; PTH, parathyroid hormone.

### 3.2. Effects of Polaprezinc Supplementation on Anemia Management

**Figure 1:**
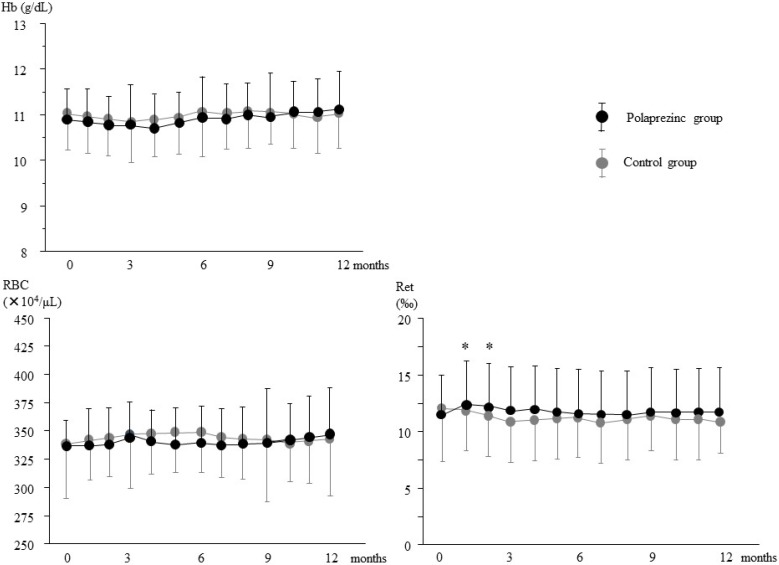
Changes in hemoglobin, red blood cell count, and reticulocyte levels during the study period. Hb, hemoglobin; RBC, red blood cell; Ret, reticulocyte. Data are expressed as mean ± SD, * *p* < 0.05 *vs.* baseline.

There were no significant changes in red blood cell (RBC) counts or Hb levels within groups or between the control and polaprezinc groups during the 12-month study period (Figure 1). A significant increase in the reticulocyte count was noted at one and two months after polaprezinc treatment compared with baseline values, although this was transient. No such findings were observed in the control group.

**Figure 2:**
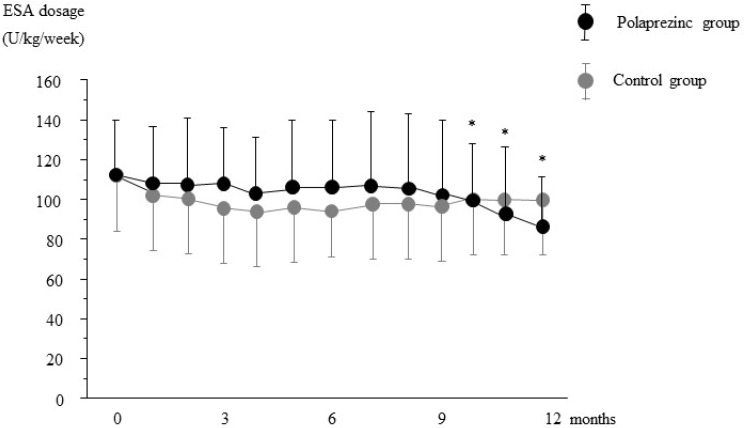
Changes in the required weekly recombinant human erythropoietin dosage during the study period. ESA, erythropoiesis-stimulating agent, Data are expressed as mean ± SD, * *p* < 0.05 *vs.* baseline.

**Figure 3:**
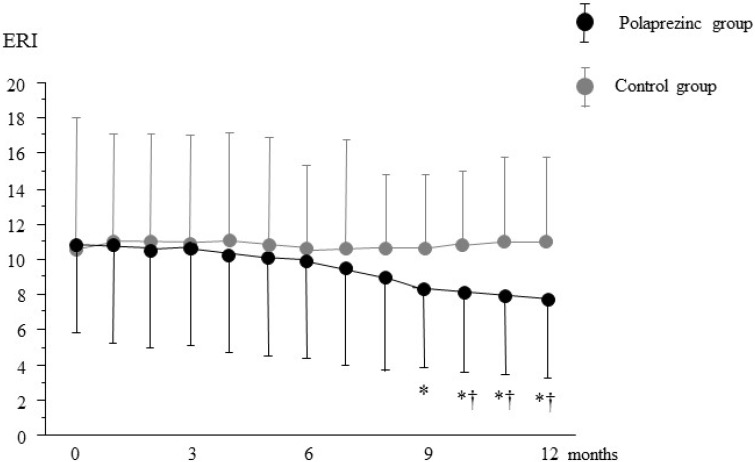
Changes in erythropoietin responsiveness index (ERI) during the study period. Data are expressed as mean ± SD, * *p* < 0.05 *vs.* baseline, † *p* < 0.05 *vs.* control group.

As shown in Figure 2, the dose of ESA was significantly decreased at 10 months after polaprezinc treatment compared to the baseline values; this was continued to the end of the study. However, there was no significant difference in ESA dose between the two groups. As shown in Figure 3, ERI was significantly decreased at nine months compared with the baseline value in the polaprezinc group; the decrease persisted until the end of the study. Furthermore, a significant difference in ERI was observed between the groups from 10 to 12 months.

### 3.3. Effects of Polaprezinc Supplementation on Mineral Parameters

As listed in Table 2, serum zinc levels were significantly increased in the polaprezinc group after three months of polaprezinc treatment; this elevated level persisted throughout the study period (normal range of serum zinc, 65–110 μg/dL). There was a significant difference in serum zinc levels in the polaprezinc group compared with the control group from three months till the end of the study. In contrast, serum copper levels were significantly decreased in the polaprezinc group after three months, and these levels persisted until the end of the study period. On the other hand, there were no significant changes in the serum zinc or copper levels in the control group. No significant differences were noted in the serum calcium or phosphate levels in the two groups.

**Table 2:** Changes in various parameters during the study period.

Variables	Group	Baseline	3 Months	6 Months	9 Months	12 Months
Serum zinc	Polaprezinc	53 ± 6	67 ± 12 ^†,^***	73 ± 21 ^‡,^***	83 ± 27 ^‡,^***	80 ± 18 ^‡,^***
	Control	55 ± 5	54 ± 6	56 ± 7	56 ± 7	56 ± 10
Serum copper	Polaprezinc	89 ± 17	83 ± 13 *	82 ± 14 *	79 ± 11 **	79 ± 12 ***
	Control	88 ± 17	86 ± 17	86 ± 17	85 ± 17	85 ± 23
Corrected calcium	Polaprezinc	8.7 ± 0.5	8.6 ± 0.7	8.7 ± 0.6	8.8 ± 0.6	8.8 ± 0.5
	Control	8.6 ± 0.4	8.7 ± 0.6	8.6 ± 0.4	8.8 ± 0.6	8.8 ± 0.6
Serum iron	Polaprezinc	59 ± 16	53 ± 19	60 ± 21	62 ± 21	57 ± 24
	Control	64 ± 30	57 ± 34	62 ± 24	57 ± 18	56 ± 23
TSAT (%)	Polaprezinc	28 ± 8	26 ± 9	28 ± 8	30 ± 15	30 ± 15
	Control	29 ± 14	28 ± 18	30 ± 13	29 ± 12	27 ± 14
Serum ferritin	Polaprezinc	142 ± 74	128 ± 53 *	121 ± 38 *	115 ± 32 *	108 ± 30 **
	Control	145 ± 85	129 ± 83	133 ± 93	141 ± 83	141 ± 83

TSAT, transferrin saturation. * *p* < 0.05, ** *p* < 0.01, ***p < 0.0001 *vs.* baseline, ^†^ *p* < 0.01, ^‡^ *p* < 0.0001 *vs.* control group.

Although there were no significant changes in the serum iron and TSAT levels in the polaprezinc group during the study period, the serum ferritin levels were significantly decreased from three months after polaprezinc treatment and continued to decrease throughout the study. On the other hand, there were no significant changes in the serum iron, TSAT, or ferritin levels in the control group. Further, the required dose of cideferron showed no significant differences between the groups.

### 3.4. Multivariate Regression Analysis

**Figure 4:**
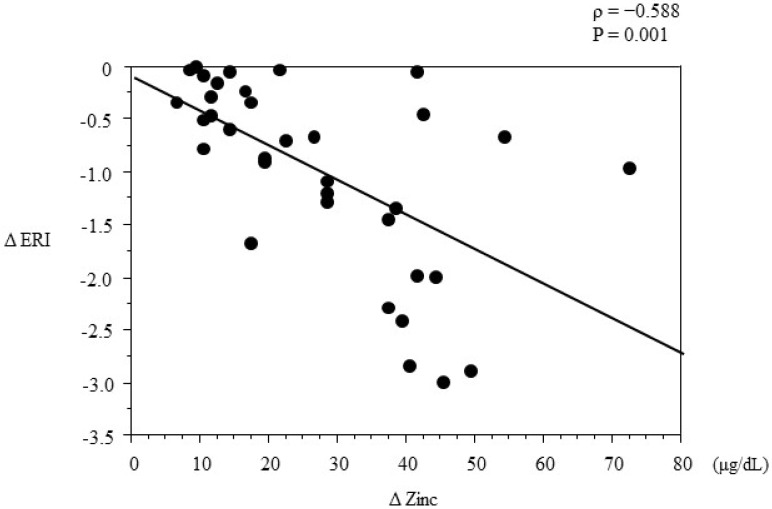
Correlation between ΔERI and Δzinc in the polaprezinc group. ΔERI was calculated as the difference between ERI at baseline and ERI at the end of the study (ERI at 12 months minus ERI at baseline), Δzinc was calculated as the difference between the serum zinc level at baseline and serum zinc level at the end of the study.

There was no significant association between the change in ERI from baseline till the end of the study (ΔERI) and any measured clinical parameter, including age, HD duration, or changes in serum iron, ferritin, and copper levels. However, there was a significant correlation between ΔERI and the change in serum zinc levels from baseline till the end of the study (Δzinc) level (*ρ* = −0.588, *p* = 0.001; Figure 4). Multivariate regression analysis was performed to identify the independent predictors of ΔERI in the polaprezinc group (Table 3). Multiple stepwise regression analysis was performed using ΔERI as the dependent variable and clinical parameters including age, sex, HD duration, the presence of diabetes, serum albumin, Hb concentrations, and mineral and iron parameters as independent variables. Only the change in serum zinc level was an independent predictor of ΔERI (*R*^2^ = 0.551).

**Table 3:** Independent predictors of change in erythropoietin responsiveness index in the polaprezinc group.

Variables	β	SE	95% CI		*p* value
			Lower	Upper	
Age	0.0070	0.0224	−0.0398	0.0546	0.7466
Hemodialysis duration	0.0010	0.0118	−0.0238	0.0259	0.9327
Male sex	−0.0450	0.1946	−0.4542	0.3637	0.8188
Diabetes	0.3140	0.3530	−0.4268	1.0657	0.3842
Kt/V	3.3530	2.7444	−2.4126	9.1190	0.2375
Baseline hemoglobin	−0.1080	0.3230	−0.7873	0.5700	0.7045
Serum albumin	−0.3800	0.6987	−1.8489	1.0872	0.5924
Change in iron	0.0001	0.0077	−0.0163	0.0163	0.9976
Change in TSAT	0.0086	0.0155	−0.0240	0.0412	0.5860
Change in ferritin	0.0011	0.0027	−0.0046	0.0070	0.6801
Change in copper	−0.0027	0.0131	−0.0303	0.0249	0.8375
Change in zinc	−0.0479	0.0120	−0.0740	−0.0219	0.0011
Calcium	−0.3040	0.3562	−1.0524	0.4443	0.4046
Phosphate	−0.4355	0.6372	−1.7743	0.9032	0.5030
Intact PTH	0.0050	0.0026	−0.0005	0.0106	0.0720
High sensitivity-CRP	1.7622	1.1604	−0.6758	4.2002	0.1462

CRP, C-reactive protein; PTH, parathyroid hormone; TSAT, transferrin saturation.

## 4. Discussion

This study suggested the following important clinical issues. First, oral zinc supplementation reduced the ERI in anemic patients with HD. Second, serum ferritin and copper levels were decreased by zinc supplementation, and the change in serum zinc level was an independent predictor of the reduction in ERI.

The first important clinical finding from this study is that oral zinc supplementation is a potential treatment for anemic patients treated with ESA. Anemia in patients with ESRD is an independent risk factor for cardiac disease and mortality [10,11,12], and hemoglobin levels are strongly correlated with the quality of life [13]. Accordingly, ESA is used as an effective treatment for anemia in HD patients. On the other hand, some reports have demonstrated that inadequate iron mobilization, infectious or non-infectious inflammatory states, and deficiency of folic acid and vitamin B12 may cause ESA hyporesponsiveness in patients with HD [3,14,15,16]. In this study, we focused on zinc deficiency in dialysis patients—caused by inadequate intake and malabsorption—and we observed a statistically significant reduction in ERI after initiating daily polaprezinc supplementation. Although no previous studies have directly demonstrated a correlation between zinc concentrations and hemoglobin production, some studies have shown that zinc finger proteins composed of zinc (e.g., growth factor independence-1B (Gfi-1B)), play an important role in erythroid differentiation and development [17]. Osawa *et al.* reported that Gfi-1B supports the proliferation of immature erythroblasts through its zinc finger domain and regulates transcription during erythropoiesis [18]. These findings strongly implicate the involvement of Gfi-1B in normal erythropoiesis and support its potential role in hematopoietic stem cells. Although the relationship between zinc finger proteins and low zinc concentrations remains unclear, we consider that the improvement in renal anemia following zinc supplementation is based on improved erythropoiesis.

The second clinical issue raised by the present findings is that serum ferritin and copper levels were decreased by zinc supplementation; moreover, the decrease in ERI was proportionate to changes in serum zinc levels from baseline till the end of the study. Further, the reticulocyte count increased briefly after initiation of polaprezinc therapy and decreased to the basal level in about three months. Another study has shown that ESA dosage can be reduced from baseline to significantly lower levels at 12 weeks despite the decrease of reticulocytes in patients receiving HD [19]. In patients with zinc and iron deficiency and ESA-hyporesponsive anemia, zinc supplementation leads to improvement of anemia in a shorter duration [19]. However, our patients required a longer duration for increasing serum zinc levels and decreasing ERI, as they had been treated with a lower ESA dosage at baseline and did not have iron deficiency. Therefore, patients with ESA-hyporesponsive anemia were not included in our study. It has also been reported that proton-pump inhibitors inhibit supplemental zinc absorption in non-uremic patients [20]. Because the subjects in the present study were all uremic patients and many of our patients had been treated with proton-pump inhibitors, the response to increased serum zinc levels and decreased ERI might be delayed.

Inflammation leads to the production of hepcidin, a hormone that regulates iron release from iron-rich cells [21]. During inflammation, hepcidin causes endocytosis of ferroportin, the “iron-export protein”, decreasing serum iron levels. These conditions lead to low TSAT levels and ultimately anemia. Ferritin is expressed in response to oxidative stress and inflammation, and is secreted into the extracellular matrix and serum [22]. In the present study, serum ferritin levels were significantly decreased without changes in serum iron and TSAT levels in the polaprezinc group, suggesting that zinc therapy enhanced the amount of iron required and hemoglobin-producing ability without requiring up-regulation of the cellular iron storage protein, ferritin. Furthermore, it has been reported that zinc supplementation improves tight junction barrier function in renal as well as gastrointestinal epithelial cells *in vitro* [23]. Thus, although zinc supplementation may act directly on the kidneys in addition to its effect on renal anemia, those effects on gastrointestinal epithelial cells are unknown for the uremic state. Further studies are therefore needed.

Because zinc, iron, and copper are all divalent metal cations, their absorption via transporters on the intestinal epithelial cells, such as divalent metal-ion transporter-1, may be antagonized [24,25]. Although serum iron levels were not decreased because of intravenous iron supplementation during HD, serum copper levels were significantly decreased after zinc supplementation. However, given that this decrease was within the normal range, the dosage of zinc at 34 mg/day appears to be safe. Since the change in serum zinc levels was an independent predictor of the reduction in ERI, zinc supplementation may be a useful therapeutic strategy for renal anemia treatment in patients undergoing HD and with low serum zinc levels.

Our study has limitations. The study was limited by the small sample size and short treatment duration. Additional long-term randomized controlled trials are needed to accurately assess the effects of zinc supplementation and evaluate the optimal dose of zinc in dialysis patients. Moreover, the relationship between zinc levels and erythrocyte maturation needs to be clarified in detail.

The incidence of chronic kidney disease is increasing worldwide, and the high cost of managing these patients is a key issue. In the United States in 2010, ESAs expenditures were a total of $1.87 billion, representing Medicare’s single largest drug expenditure among patients on dialysis and constituting 67% of all Medicare spending on injectables by this group. [26]. Thus, it is important to identify less expensive methods for the appropriate management of HD patients. Accordingly, the present study is meaningful in its assessment of zinc supplementation as a cost-effective therapy. Some reports have shown other physiological actions of zinc, including its roles as a neurotransmission factor [27] and antioxidant [28], and further studies are needed.

## 5. Conclusions

In conclusion, zinc supplementation reduces ERI in patients undergoing HD and may be a novel therapeutic strategy for patients of renal anemia with low serum zinc levels.
